# Effects of dietary Bopu powder supplementation on intestinal development and microbiota in broiler chickens

**DOI:** 10.3389/fmicb.2022.1019130

**Published:** 2022-10-13

**Authors:** Yang Liu, Qinjin Wang, Hua Liu, Jiax‑ing Niu, Ning Jiao, Libo Huang, Shuzhen Jiang, Qinglin Guan, Weiren Yang, Yang Li

**Affiliations:** ^1^Shandong Provincial Key Laboratory of Animal Biotechnology and Disease Control and Prevention, Department of Animal Science and Veterinary Medicine, Shandong Agricultural University, Tai’an, China; ^2^Shandong Wonong Agro-Tech Group Co., Ltd., Weifang, China; ^3^College of Animal Science and Technology, Hunan Agriculture University, Changsha, China; ^4^Shandong Landoff Biotechnology Co., Ltd., Tai’an, China

**Keywords:** broiler, Bopu powder, intestinal development, microbiota, plant extract

## Abstract

This study aimed to investigate the effect of dietary supplementation with Bopu powder on intestinal development and bacterial community composition in broiler chickens. A total of 486 1-day-old arbor acres broilers were fed a basal diet (CON group), a basal diet supplemented with 50 mg/kg aureomycin (AB group), or a basal diet supplemented with 40 mg/kg Bopu powder (BP group). The results showed that the BP group had significantly lower serum tumor necrosis factor-α (TNF-α), interleukin (IL)-1β, IL-6, and diamine oxidase concentrations and had significantly higher serum IL-10 concentrations than CON group (*p* < 0.05). Groups AB and BP had a significantly higher weight per unit length of the small intestine and villus height than the CON group (*p* < 0.05), and BP group had a significantly higher ratio of villus height to crypt depth than groups CON and AB (*p* < 0.05). Compared to the CON group, dietary Bopu powder or aureomycin supplementation significantly increased transforming growth factor-α concentration and mRNA expressions of zonula occludens-1 (ZO-1) and occludin, and decreased intestinal mucosal concentrations of TNF-α, IL-6, IL-10, caspase-3, and caspase-8 and mRNA expressions of nuclear factor-kappa-B and *Bax*/*Bcl-2* ratio in the intestinal mucosa (*p* < 0.05). Meanwhile, BP group had significantly higher ZO-1, secretory immunoglobulin A, interferon-γ concentrations, and mRNA expressions of glucose transporter type-2 and sirtuin-1, and significantly lower IL-1β concentration than groups CON and AB in intestinal mucosa (*p* < 0.05). Dietary Bopu powder supplementation significantly increased the concentration of trefoil factor family member and mRNA expressions of superoxide dismutase-1 and bcl-2 associated X, and significantly reduced casepase-9 concentration and myeloid differentiation primary response-88 expression in the intestinal mucosa of broiler chickens relative to CON group (*p* < 0.05). Moreover, results of high-throughput sequencing showed that broilers in the BP group had microbial community structure distinct from that in CON group, and the addition of Bopu powder increased the abundances of *Faecalibacterium* and *Colidextribacter* (*p* < 0.05). Therefore, our study suggests a synergic response of intestinal development and microbiota to the Bopu powder, and provides a theoretical basis as a potential substitute for antibiotics.

## Introduction

The intestinal epithelium facilitates selective uptake of nutrients while serving as a barrier against harmful pathogens (Beumer and Clevers, 2021). In poultry production, intestinal mucosal injury is a prevalent disease in broiler chickens (Kogut et al., 2018). When the intestinal barrier is damaged, it will increase the transport of endotoxins and pathogens, resulting in serious damage to the body (Gonzalez-Gonzalez et al., 2018; Beumer and Clevers, 2021). Moreover, the intestinal tract is rich in microorganisms or flora, which is crucial to intestinal development and maintaining intestinal physiological function homeostasis (Diaz Carrasco et al., 2019). Since their discovery and application, antibiotics have played an unparalleled role in livestock and poultry production, and shown a protective effect in development and function of small intestine (Hafeez et al., 2020). However, antibiotic resistance has become a serious threat to human beings and the environment because of the irrational use of antibiotics (Cheng et al., 2014). Nowadays, with the banning of antibiotics, it has become the primary task to find antibiotic substitutes, especially natural feed additives.

An accumulating body of research indicates *Macleaya Cordata* extract (MCE) has antibacterial, anti-inflammatory, insecticidal, anti-tumor and other biological activities (Lin et al., 2018), and is a good substitute for antimicrobial growth promoter (AGP; Zhang et al., 2021). The main active components of MCE include benzophenanthridine alkaloids (sanguinarine and chelerythrine) and isoquinoline alkaloids (proto alkaloids and allocrine alkaloids; Kosina et al., 2010). In 2005, compounds containing sanguinarine and chelerythrine were used as feed additives in the European Union (Xie et al., 2015). Recently, the components made up of protopine and allocryptopine have been registered as veterinary drugs in China, and named Bopu powder (Veterinary Drug No. 180415374; Liu H. et al., 2022). Liu H. et al. (2022) showed that supplementation with 25–50 mg/kg Bopu powder in the diet improved antioxidant capacity and increased the abundances of beneficial bacteria in foregut of laying hens. Our recent study demonstrated that the growth performance and liver health of broiler chickens could be improved by adding protopine and allocryptopine (Bopu powder) to the diet (Liu et al., 2022a). However, there is little literature evaluating the effects of dietary Bopu powder supplemented on intestinal development and microbiota community structures of broiler chickens.

Therefore, the purpose of this study was to explore the effects of dietary supplemented with Bopu powder on intestinal development and microbiota community structures of broiler chickens, and to evaluate the potential applications of Bopu powder as an effective substitute to AGP.

## Meterials and methods

### Experimental design and management

In the 42-day study, 486 newly hatched male Arbor Acres (AA) broilers with an average body weight (BW) of 48.76 ± 0.25 g were selected. The broilers were purchased from Shandong Fengxiang Co. Ltd. (Liaocheng, China) and randomly allocated into three dietary treatments with six replicates per treatment and 27 broilers per replicate. Broilers in each replicate were housed in a three-level cage, and all cages were placed in a temperature- and light-controlled room with continuous light in the experimental farm of Shandong Agricultural University. Three treatment groups were as follows: (1) the control (CON) group, which was fed a basal diet; (2) the AB group, which was fed a basal diet supplemented with 50 mg/kg aureomycin; and (3) the BP group, which was fed a basal diet supplemented with 40 mg/kg Bopu powder (Liu et al., 2022a). The 42-day experiment was conducted with starter phase (day 0–21) and the growth phase (day 21–42). The basal diets (Supplementary Table S1) were formulated to meet the nutritional requirements of the Ministry of agriculture of China (2004). The Bopu powder was provided by the Micolta Bioresource Company Limitid (Changsha 410331, China), and made up of 1% protopine, 0.5% allotypotopine, and 98.5% starch (Liu H. et al., 2022). All management was carried out according to the AA Broiler Management Guide (Acres, 2009), and fresh feed and water were provided every day for broiler chickens during the experiment. The temperature of room was maintained at 35°C for the first week and then gradually reduced by 1°C every 2 days until it reached 21°C.

### Sample collection

After a 12-h fast on the last day of the experiment, a broiler with close to the average body weight was selected in each replicate. Following the collection of blood samples (5 ml) from the wing vein, serum was obtained by centrifugation at 3,000 rpm/min for 15 min at 4°C and stored at −20°C until analysis. After the intestine of each broiler chicken was quickly removed, intestinal length and weight were measured, and the relative index was calculated. Then a 2-cm length segment was cut from the middle part of small intestine, washed with the 0.9% saline solution, and fixed with 4% paraformaldehyde solution. Subsequently, the mucosal tissue was scraped with sterile glass slide from the same parts of small intestine after being washed with ice-cold saline solution, quick-frozen in liquid nitrogen, and finally stored at −80°C until further analysis. In addition, cecal contents were quickly collected with sterile fecal collection tubes and stored at −80°C for microbiological analysis.

### Intestinal morphology observation

The small intestine tissue was taken out after being fixed with paraformaldehyde for 24 h, and embedded according to the conventional paraffin embedding scheme (Xiao et al., 2021). Then the tissues were sliced into 5-um thickness with automatic slicer (HM355S, Burton International Trading Co., Ltd., China), stained with hematoxylin–eosin, and sealed with neutral resin. The morphology of small intestine was observed and photographed with a Nikon Elipse 80i microscope (Nikon, Tokyo, Japan). A total of 10 well oriented crypt-villus units per sample were chosen to measure villus height (VH) and crypt depth (CD) using image analysis software (JEDA, Nanjing, Jiangsu, China), and the ratio of VH to CD was calculated (Chen et al., 2021a).

### Analysis of serum inflammatory factors

The levels of tumor necrosis factor-α (TNF-α), interleukin-1β (IL-1β), interleukin-6 (IL-6), and interleukin-10 (IL-10) in the serum of broiler chickens were assayed with ELISA kits (Jiangsu Meimian Industrial Co., Ltd.) as described in a previous study (Chen et al., 2021a).

### Analysis of serum DAO and D-lactate levels

The serum diamine oxidase (DAO) activity and D-lactate concentrations were analyzed by chicken-specific kits (Jiangsu Meimian) according to the determination steps of ELISA operation described in Li et al. (2022).

### Analysis of intestinal barrier-related function

The zonula occluden-1 (ZO-1), mucin 2 (MUC2), trefoil factor family member (TFF), and transforming growth factor-α (TGF-α) in the intestines were examined using ELISA kits (Jiangsu Meimian) following the protocol described previously (Li et al., 2022).

### Analysis of SIgA and intestinal inflammatory factors

The levels of secretory immunoglobulin A (SIgA), tumor necrosis factor-α (TNF-α), interleukin-1β (IL-1β), interleukin-6 (IL-6), interleukin-10 (IL-10), and interferon-γ (IFN-γ) in the intestinal samples of broiler chickens were assayed using ELISA kits (R&D Systems Inc., Minneapolis, MN, United States). The determination steps were followed with the standardized ELISA procedures described in a previous study (Chen et al., 2021a; Liu et al., 2022b).

### Analysis of intestinal caspases activities

The activities of caspase-3, caspase-8, and caspase-9 in intestinal samples of broiler chickens were determined using chicken-specific ELISA kits (Beyotime Biotech) followed with the standardized ELISA procedures described by Chen et al. (2021a).

### Analysis of gene expression

Briefly, 50–100 mg of intestinal tissue samples were taken and placed in a mortar containing liquid nitrogen, and ground to a powder. Then 1 ml of TRIzol-reagent was added to extract total RNA. Subsequently, RNA was reverse transcribed to cDNA by Evo M-MLV RT Kit (Accurate Biotechnology, Hunan, China) according to the kit manual. The mRNA expression levels of zonula occludens-1 (*ZO-1*), occludin (*OCLN*), claudin-2 (*CLND2*), claudin-3 (*CLND3*), glucose transporter type 2 (*GLUT2*), sodium-glucose transporter 1 (*SGLT1*), y + L amino acid transporter-1 (*y + LAT1*), cationic amino acid transporter-1 (*CAT1*), fatty acid binding protein-1 (*FABP1*), Toll-like receptor 4 (*TLR4*), myeloid differentiation factor 88 (*MyD88*), nuclear factor-κB (*NF-κB*), sirtuin1 (*Sirt1*), nuclear factor erythroid 2-related factor 2 (*Nrf2*), heme-oxygenase 1 (*HO-1*), catalase (*CAT*), superoxide dismutase 1 (*SOD1*), superoxide dismutase 2 (*SOD2*), glutathione peroxidase-1 (*GPX1*), NAD(P)H quinone oxidoreductase 1 (*NQO1*), bcl-2 associated X (*Bax*), and b-cell lymphoma-2 (*Bcl-2*) in intestinal samples were assessed using LightCycler 96 (Roch, Switzerland) with SYBR® Green Premix Pro Taq HS qPCR Kit (AG11701, Accurate Biology, Da Lian, China) as described previously (Liu et al., 2022a). *β*-actin was used as an internal reference, and 2^-ΔΔCT^ method was used to calculate the relative expressions of target genes. All gene primers are shown in Supplementary Table S2.

### Microbial analysis

Total genomic DNA was extracted from frozen fecal samples using QIAamp DNA·Stool Mini Kits (Qiagen Inc., Hilden, Germany) according to the manufacturer’s protocol. Depending on the concentration, DNA was diluted to 1 ng/μl using sterile solution, and the V4 hypervariable region of 16S rDNA was amplified using 515f and 806r primers (5′-GTGCCAGCMGCCGCGGTAA-3′ and 5′-GGACTACHVGGGTWTCTAAT-3′, respectively; Chen et al., 2021b). Subsequently, TruSeq ® DNA PCR-Free Sample Preparation Kit (Illumina, United States) was used to generate sequencing libraries, and the library quality was assessed on the Qubit@2.0 fluorometer (Thermo Scientific) and Agilent Bioanalyzer 2100 system. At last, the library was sequenced on the Illumina NovaSeq platform and 250 bp paired-end reads were generated. Paired-end reads was assigned to samples based on their unique barcode and truncated by cutting off the barcode and primer sequence. After sequence assembly, data filtration and chimera removal, the final effective sequences were obtained. The sequences with ≥97% similarity were allocated to the same OTU through Uparse software, and then each sequence was labeled with the Silva Database based on the Mothur algorithm to classify it to different classification levels (Quast et al., 2012). Alpha diversity was applied in analyzing complexity of species diversity for a sample through 4 indexes, including Shannon, Simpson, Chao1, and ACE. All these indicators of samples were calculated with QIIME software (Version 1.9.1) and displayed with R software (Version 2.15.3; Lawley and Tannock, 2017). Beta diversity based on bray_curtis distance was calculated using QIIME software. Principal coordinate analysis (PCoA) was selected to calculate and visualize the unifrac distance. The unweighted pair-group method with arithmetic mean (UPGMA) clustering analysis with the bray-curtis distance was also used to visualize the dissimilarity matrices of OTUs (Lozupone and Knight, 2005).

### Statistical analysis

The data were statistically analyzed using one-way ANOVA of SAS 9.4 (Institute Inc., Cary, NC, United States). Multiple comparisons of treatment means were examined using the least significant difference test. Spearman’s correlation was used to evaluate the associations between differential bacterial abundances and concentrations of metabolic parameters and immunological markers in serum and intestine. All data were presented as the mean ± standard error. The values of *p* < 0.05 were regarded as statistically significant, and the values of 0.05 ≤ *p* < 0.10 were considered as a significance trend.

## Results

### Effects of Bopu powder on serum inflammatory factors, DAO, and D-lactate concentrations in broiler chickens

**Figure 1:**
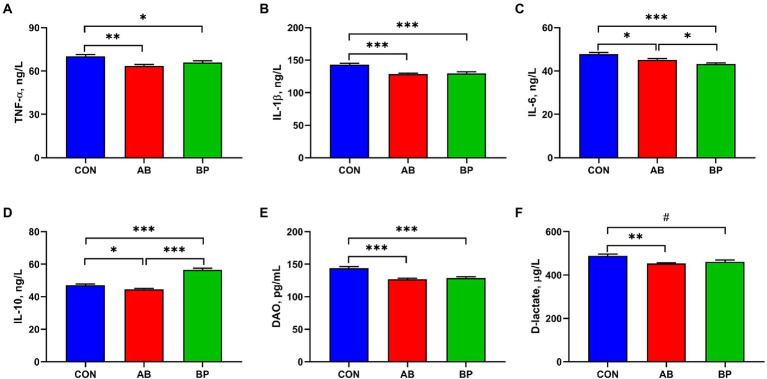
Effects of dietary supplemented with Bopu powder on serum inflammatory factors, diamine oxidase (DAO), and D-lactate in broiler chickens. **(A)** Tumor necrosis factor-α (TNF-α); **(B)** Interleukin-1β (IL-1β); **(C)** Interleukin-6 (IL-6); **(D)** Interleukin-10 (IL-10); **(E)** DAO; and **(F)** D-lactate. CON group, broiler chickens fed a basal diet; AB group, broiler chickens fed a basal diet supplemented with 50 mg/kg aureomycin; and BP group, broiler chickens fed a basal diet supplemented with 40 mg/kg Bopu powder containing protopine and allocryptopine. Values are mean ± SE (*n* = 6). ^#^0.05 ≤ *p* < 0.10, ^*^p < 0.05, ^**^p < 0.01, ^***^p < 0.001.

The effects of Bopu powder on serum inflammatory factors, DAO and D-lactate concentrations of broiler chickens are shown in Figure 1. Compared with CON group, serum TNF-α, IL-1β, IL-6, and DAO concentrations were significantly lower in AB group and BP group (*p* < 0.05), and BP group showed significantly lower serum IL-6 concentration than AB group (*p* < 0.05). Serum IL-10 concentration was significantly higher in BP group than in CON group and AB group (*p* < 0.05), and AB group showed significantly lower serum IL-10 concentration than CON group (*p* < 0.05). Serum D-lactate concentration was significantly lower in AB group than in CON group, and the D-lactate concentration of BP group tended to be lower than that of AB group (*p* < 0.10).

### Effect of Bopu powder on intestinal development in broiler chickens

**Figure 2:**
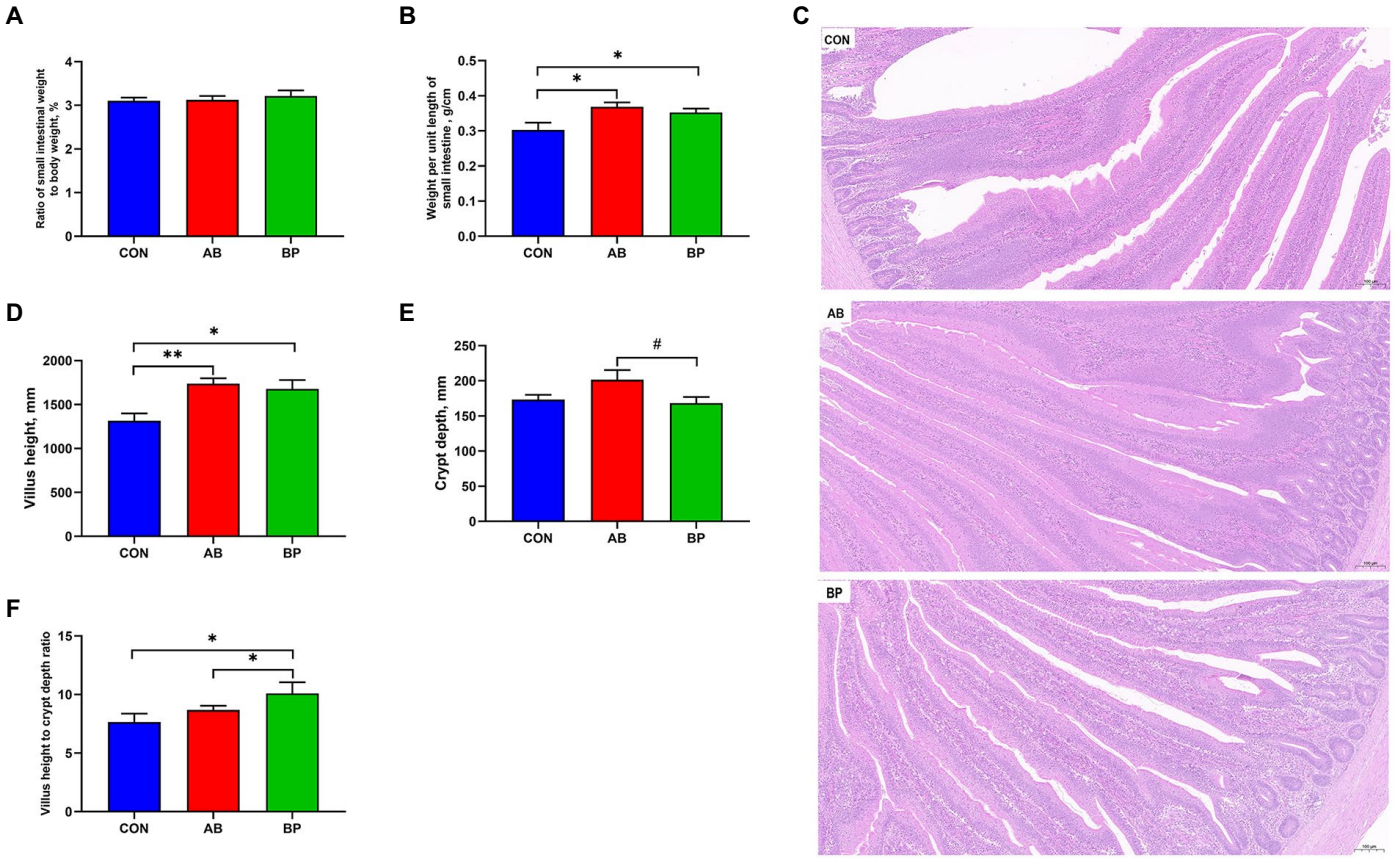
Effects of dietary supplemented with Bopu powder on intestinal development in broiler chickens. **(A)** Ratio of small intestinal weight to body weight; **(B)** Weight per until length of small intestine; **(C)** Representative images (×100) were stained with hematoxylin and eosin; **(D)** Villus height; **(E)** Crypt depth; and **(F)** Villus height to crypt depth ratio. CON group, broiler chickens fed a basal diet; AB group, broiler chickens fed a basal diet supplemented with 50 mg/kg aureomycin; and BP group, broiler chickens fed a basal diet supplemented with 40 mg/kg Bopu powder containing protopine and allocryptopine. Values are mean ± standard error (*n* = 6). ^#^0.05 ≤ *p* < 0.10, ^*^p < 0.05, ^**^p < 0.01.

The effect of Bopu powder on intestinal development of broiler chickens is shown in Figure 2. Compared with CON group, weight per unit length and VH of small intestine were significantly higher in AB group and BP group (*p* < 0.05). The VH to CD ratio in BP group was significantly higher than that in AB group and CON group (*p* < 0.05). Compared with AB group, the intestinal CD of BP group tended to be lower (*p* < 0.10). The VH to CD ratio was significantly higher in BP group than in AB group and CON group (*p* < 0.05). There was no significant difference in the ratio of small intestinal weight to body weight among three groups (*p* > 0.05).

### Effect of Bopu powder on intestinal barrier function in broiler chickens

**Figure 3:**
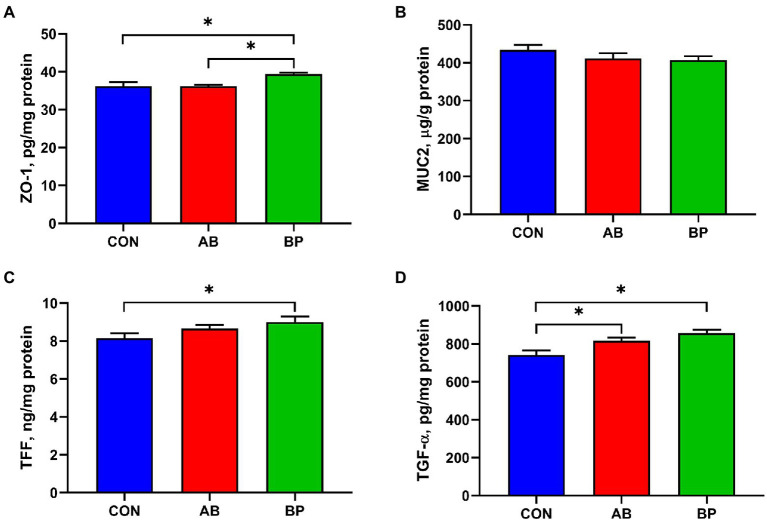
Effects of dietary supplemented with Bopu powder on intestinal barrier factors in broiler chickens. **(A)** Zonula occludens-1 (ZO-1); **(B)** Recombinant mucin 2 (MUC2); **(C)** Trefoil peptides (TFF); and **(D)** Transforming growth factor alpha (TGF-α). CON group, broiler chickens fed a basal diet; AB group, broiler chickens fed a basal diet supplemented with 50 mg/kg aureomycin; and BP group, broiler chickens fed a basal diet supplemented with 40 mg/kg Bopu powder containing protopine and allocryptopine. Values are mean ± standard error (*n* = 6). ^*^p < 0.05.

The effect of Bopu powder on intestinal barrier function in broiler chickens is shown in Figure 3. The concentration of intestinal ZO-1 was significantly higher in BP group than in AB group and CON group (*p* < 0.05). Intestinal TFF level in BP group was significantly higher than that in CON group (*p* < 0.05). Compared with CON group, BP group and AB group had significantly higher TGF-α concentration (*p* < 0.05). There was no significant difference in intestinal MUC2 concentration among three groups (*p* > 0.05).

### Effect of Bopu powder on intestinal SIgA and inflammatory factors concentrations in broiler chickens

**Figure 4:**
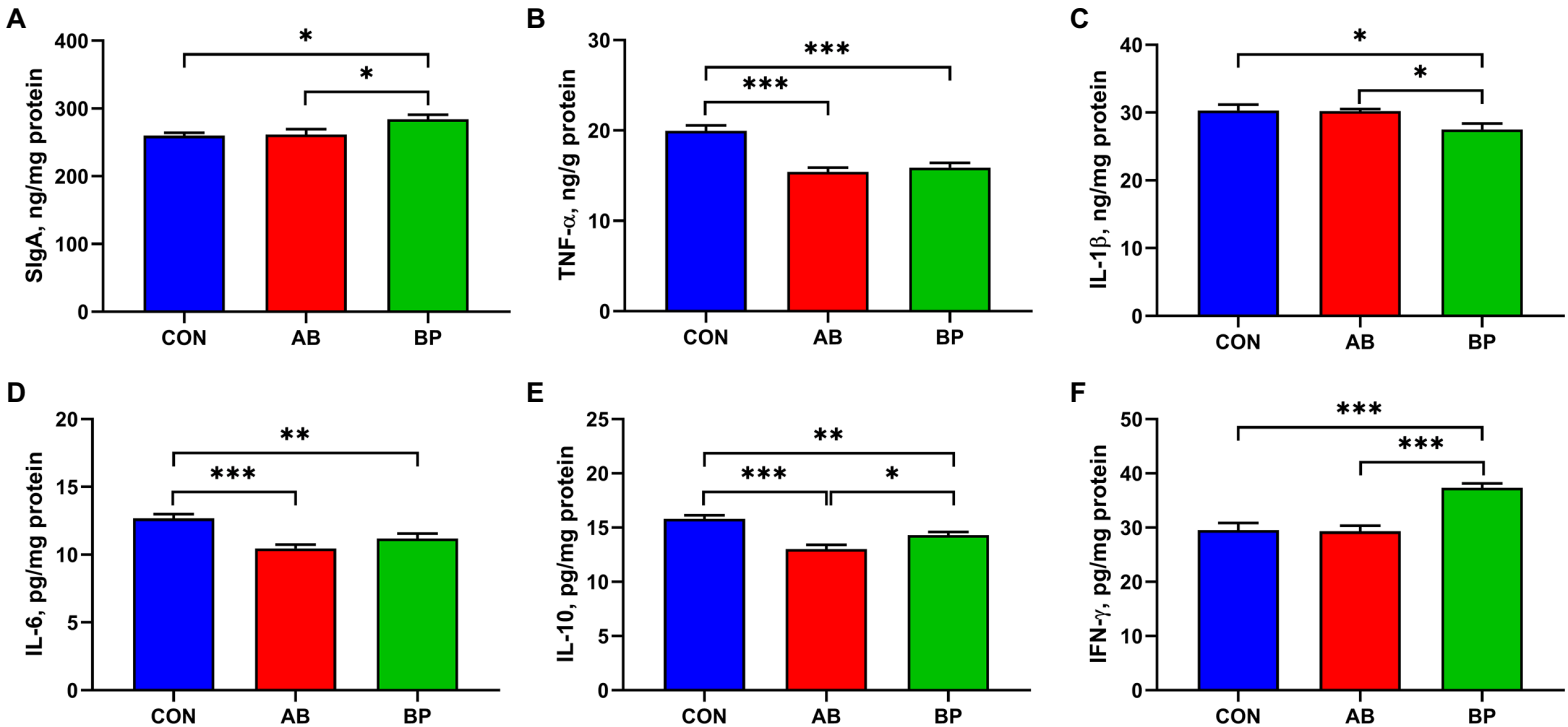
Effects of dietary supplemented with Bopu powder on Intestinal SIgA, inflammatory factors in broiler chickens. **(A)** Secretory immunoglobulin A (SIgA); **(B)** Tumor necrosis factor-α (TNF-α); **(C)** Interleukin-1β (IL-1β); **(D)** Interleukin-6 (IL-6); **(E)** Interleukin-10 (IL-10); and **(F)** Interferon-γ (IFN-γ). CON group, broiler chickens fed a basal diet; AB group, broiler chickens fed a basal diet supplemented with 50 mg/kg aureomycin; and BP group, broiler chickens fed a basal diet supplemented with 40 mg/kg Bopu powder containing protopine and allocryptopine. Values are mean ± standard error (*n* = 6). ^*^p < 0.05, ^**^p < 0.01, ^***^p < 0.001.

As shown in Figure 4, the intestinal concentrations of SIgA and IFN-γ in BP group were significantly higher than those in CON group and AB group (*p* < 0.05). The concentrations of TNF-α and IL-6 were significantly lower in AB group and BP group than in CON group (*p* < 0.05). The concentration of intestinal IL-1β was significantly higher in BP group than in AB group and CON group (*p* < 0.05). Compared with the broilers in CON group, broilers in BP group and AB group had significantly lower IL-10 concentration (*p* < 0.05), and IL-10 concentration in AB group was significantly lower than that in BP group (*p* < 0.05).

### Effect of Bopu powder on intestinal caspase activity in broiler chickens

**Figure 5:**
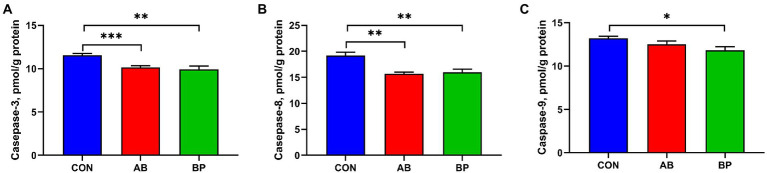
Effects of dietary supplemented with Bopu powder on caspases levels of broiler chickens. **(A)** Caspase-3; **(B)** Caspase-8; and **(C)** Caspase-9. CON group, broiler chickens fed a basal diet; AB group, broiler chickens fed a basal diet supplemented with 50 mg/kg aureomycin; BP group, broiler chickens fed a basal diet supplemented with 40 mg/kg Bopu powder containing protopine and allocryptopine. Values are mean ± standard error (*n* = 6). *p < 0.05, **p < 0.01, ***p < 0.001.

The effects of Bopu powder on intestinal caspases activities of broiler chickens are shown in Figure 5. Compared with CON group, BP group and AB group had significantly lower intestinal activities of casepase-3 and casepase-8 (*p* < 0.05). Broilers in BP group had significantly lower casepase-9 activity than broilers in CON group (*p* < 0.05).

### Effect of Bopu powder on gene expression related to intestinal barrier function in broiler chickens

**Figure 6:**
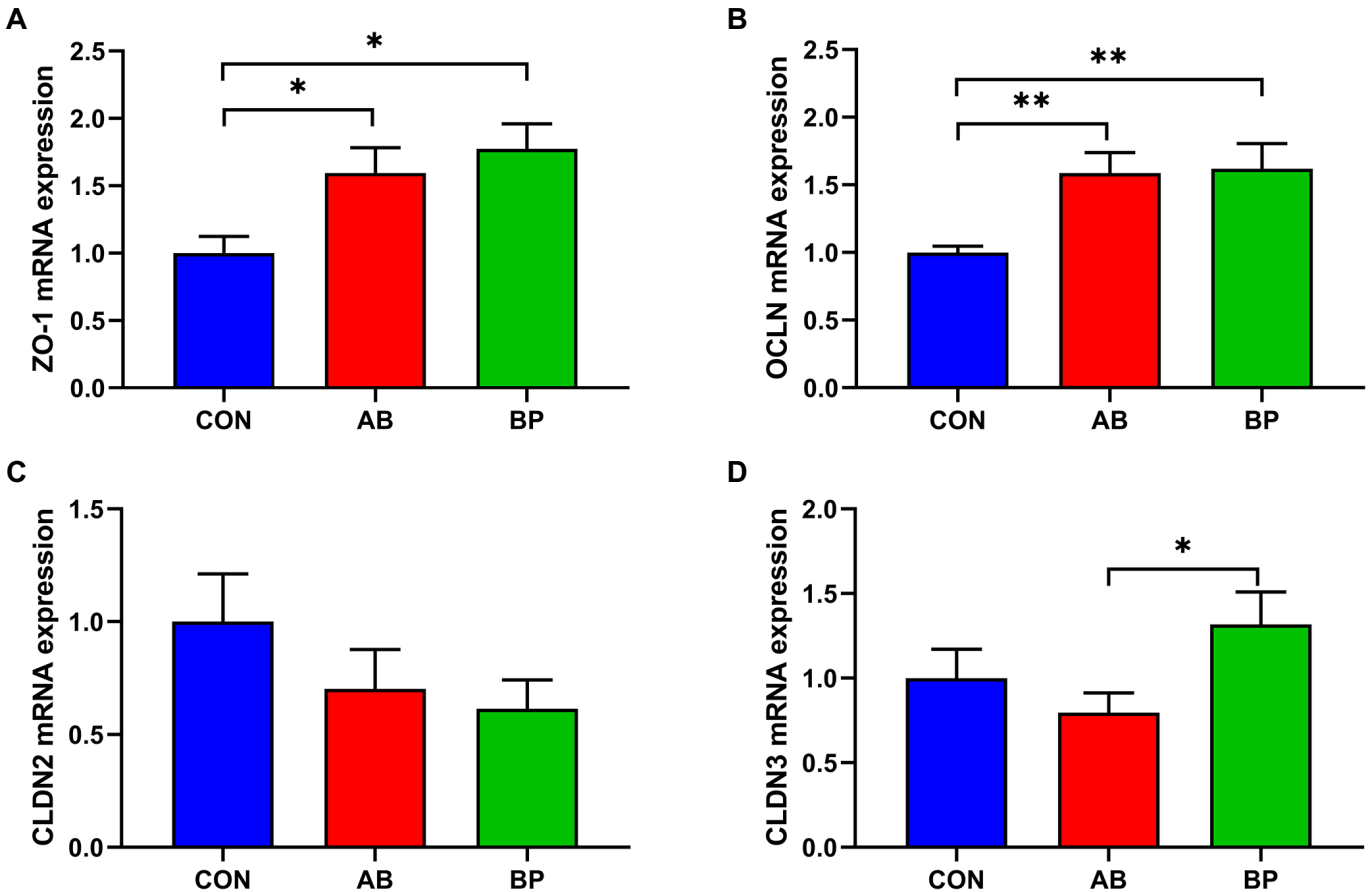
Effects of dietary supplemented with Bopu powder on expressions of barrier genes in intestine of broiler chickens. **(A)** zonula occludens-1 (*ZO-1*); **(B)** occludin (*OCLN*); **(C)** claudin-2 (*CLDN2*); and **(D)** claudin-3 (*CLDN3*). CON group, broiler chickens fed a basal diet; AB group, broiler chickens fed a basal diet supplemented with 50 mg/kg aureomycin; BP group, broiler chickens fed a basal diet supplemented with 40 mg/kg Bopu powder containing protopine and allocryptopine. Values are mean ± standard error (*n* = 6). *p < 0.05, **p < 0.01.

The effects of Bopu powder on genes expressions related to intestinal barrier function in broiler chickens are shown in Figure 6. Compared with CON group, the mRNA expressions of *ZO-1* and *OCLN* were significantly higher in AB group and BP group (*p* < 0.05). Intestinal *CLDN3* mRNA expression in BP group was significantly higher than that in AB group. No significant difference was observed in *CLDN2* mRNA expression among three groups (*p* > 0.05).

### Effect of Bopu powder on genes expressions of nutrient transporters in broiler chickens

**Figure 7:**
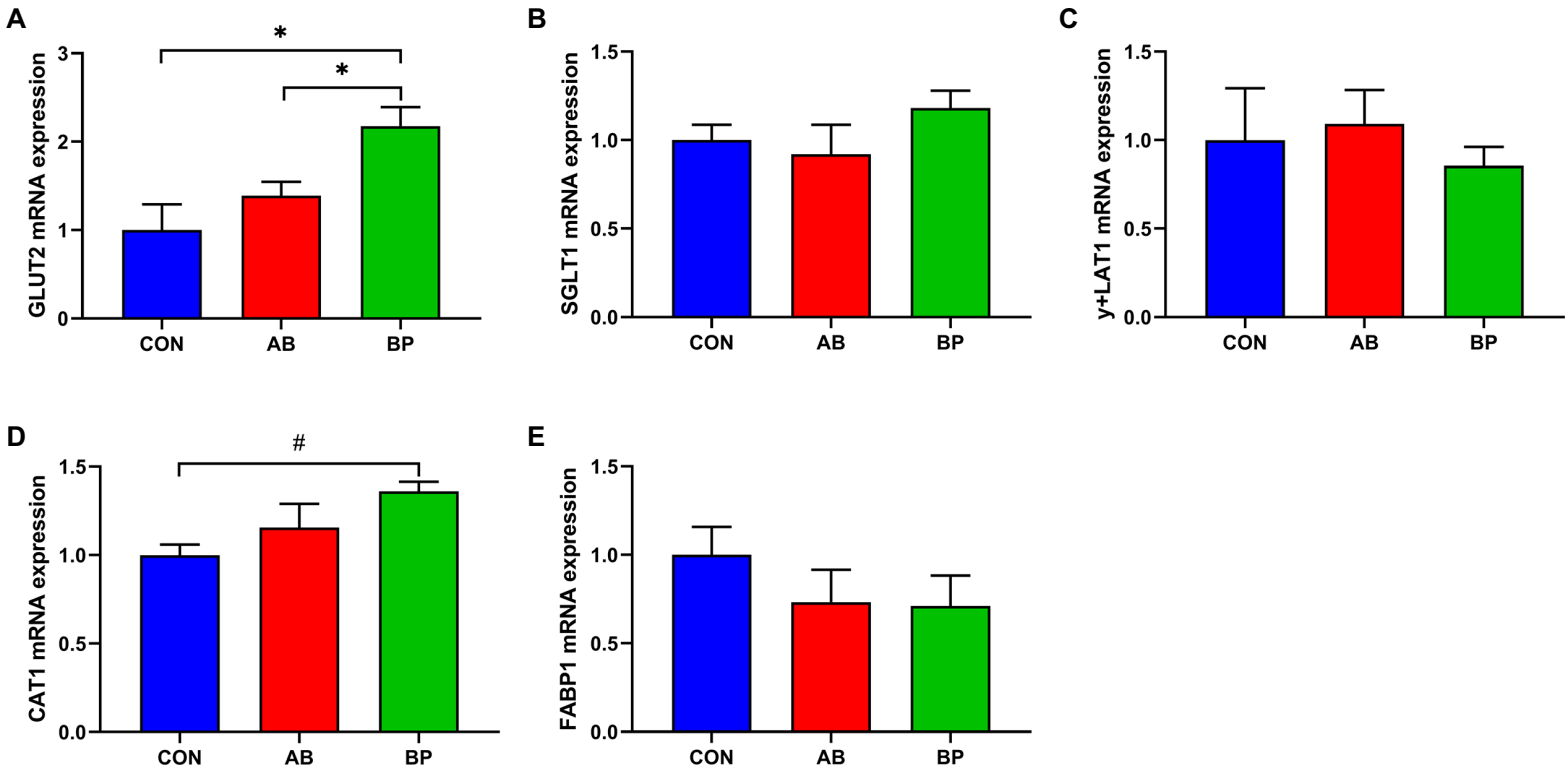
Effects of dietary supplemented with Bopu powder on expressions of nutrient transport genes in intestine of broiler chickens. **(A)** Glucose transporter type 2 (*GLUT2*); **(B)** Sodium-glucose transporter 1 (*SGLT1*); **(C)** y + L amino acid transporter-1 (*y + LAT1*); **(D)** Cationic amino acid transporter-1 (*CAT1*); and **(E)** Fatty acid binding protein-1 (*FABP1*). CON group, broiler chickens fed a basal diet; AB group, broiler chickens fed a basal diet supplemented with 50 mg/kg aureomycin; BP group, broiler chickens fed a basal diet supplemented with 40 mg/kg Bopu powder containing protopine and allocryptopine. Values are mean ± standard error (*n* = 6). ^#^0.05 ≤ *p* < 0.10, *p < 0.05.

As shown in Figure 7, the mRNA expression of *GLUT2* was significantly higher in BP group and AB group than in CON group (*p* < 0.05), and the mRNA expression of *CAT1* in BP group tended to be higher than that in CON group (*p* < 0.10). However, no significant differences were observed in expressions of *SCLT1*, *y + LAT1*, and *FABP1* among the three groups (*p* > 0.05).

### Effect of Bopu powder on intestinal inflammatory gene in broiler chickens

**Figure 8:**
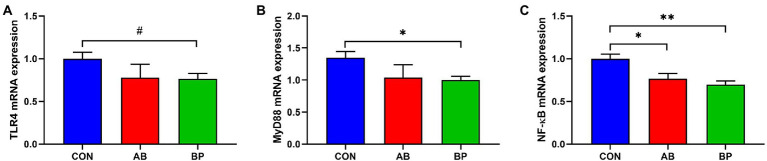
Effects of dietary supplemented with Bopu powder on expressions of inflammatory genes in intestine of broiler chickens. **(A)** Toll-like receptor 4 (*TLR4*); **(B)** Myeloid differentiation primary response 88 (*MyD88*); and **(C)** Nuclear factor-kappa B (*NF-κB*). CON group, broiler chickens fed a basal diet; AB group, broiler chickens fed a basal diet supplemented with 50 mg/kg aureomycin; BP group, broiler chickens fed a basal diet supplemented with 40 mg/kg Bopu powder containing protopine and allocryptopine. Values are mean ± standard error (*n* = 6). ^#^0.05 ≤ *p* < 0.10, *p < 0.05, **p < 0.01.

As shown in Figure 8, the mRNA expressions of *MyD88* and *NF-κB* were significantly lower in BP group than in CON group (*p* < 0.05), and the mRNA expression of *NF-κB* in AB group was significantly lower than that in CON group (*p* < 0.05). Meanwhile, the mRNA expression of *TLR4* in BP group tended to be lower than that in CON group (*p* < 0.10).

### Effects of Bopu powder on intestinal antioxidant genes expressions in broiler chickens

**Figure 9:**
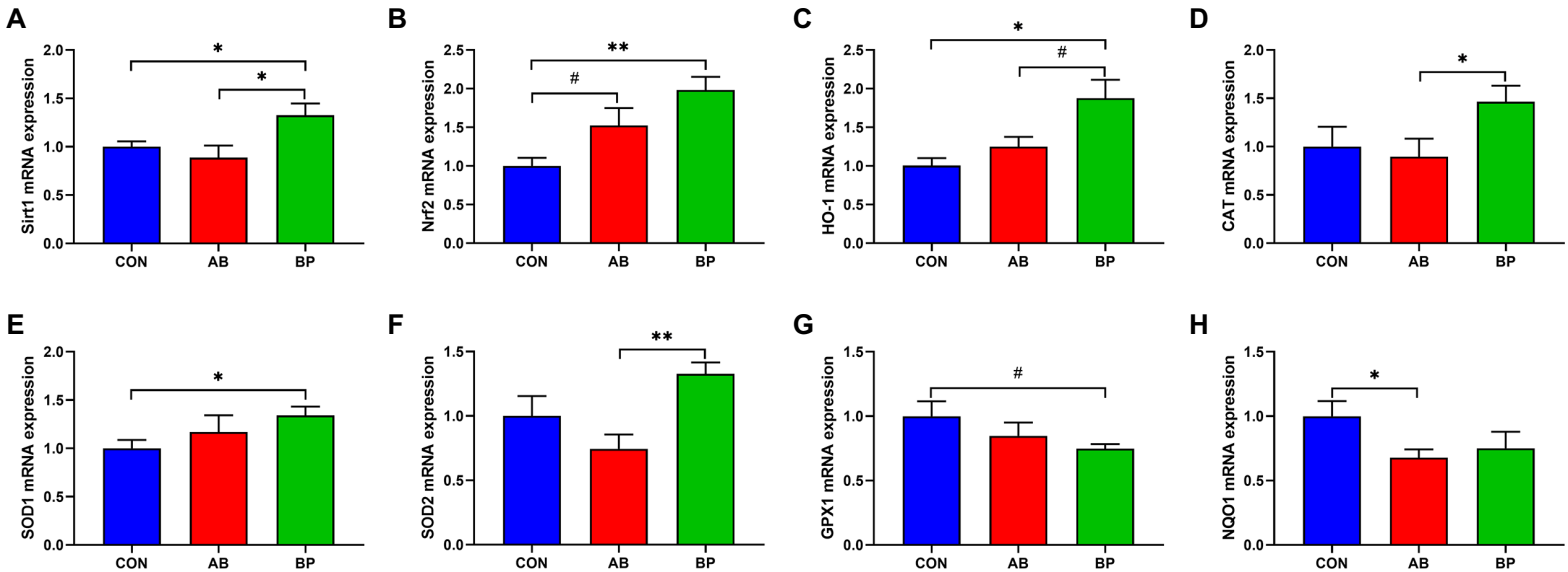
Effects of dietary supplemented with Bopu powder on expressions of antioxidant genes in intestine of broiler chickens. **(A)** Sirtuin1 (*Sirt1*); **(B)** Nuclear factor erythroid 2-related factor 2 (*Nrf2*); **(C)** Heme-oxygenase 1 (*HO-1*); **(D)** Catalase (*CAT*); **(E)** Superoxide dismutase 1 (*SOD1*); **(F)** Superoxide dismutase 2 (*SOD2*); **(G)** glutathione peroxidase-1 (*GPX1*); and **(H)** NAD(P)H quinone oxidoreductase 1 (*NQO1*). CON group, broiler chickens fed a basal diet; AB group, broiler chickens fed a basal diet supplemented with 50 mg/kg aureomycin; BP group, broiler chickens fed a basal diet supplemented with 40 mg/kg Bopu powder containing protopine and allocryptopine. Values are mean ± standard error (*n* = 6). ^#^0.05 ≤ *p* < 0.10, *p < 0.05, **p < 0.01.

As shown in Figure 9, the mRNA expressions of *Sirt1*, *Nrf2*, *HO-1*, and *SOD1* were significantly higher in BP group than in CON group (*p* < 0.05), and *Nrf2* mRNA expression in AB group tended to be higher than that in CON group (*p* < 0.10). The mRNA expression of *HO-1* in BP group tended to be higher than that in AB group (*p* < 0.10), and BP group showed significantly higher *Sirt1*, *CAT*, and *SOD2* mRNA expressions than AB group (*p* < 0.05). Besides, the *GPX1* mRNA expression of BP group tended to be lower than that of CON group (*p* < 0.10), and AB group showed significantly lower *NQO1* mRNA expression than CON group (*p* < 0.05).

### Effects of Bopu powder on intestinal apoptosis genes in broiler chickens

**Figure 10:**
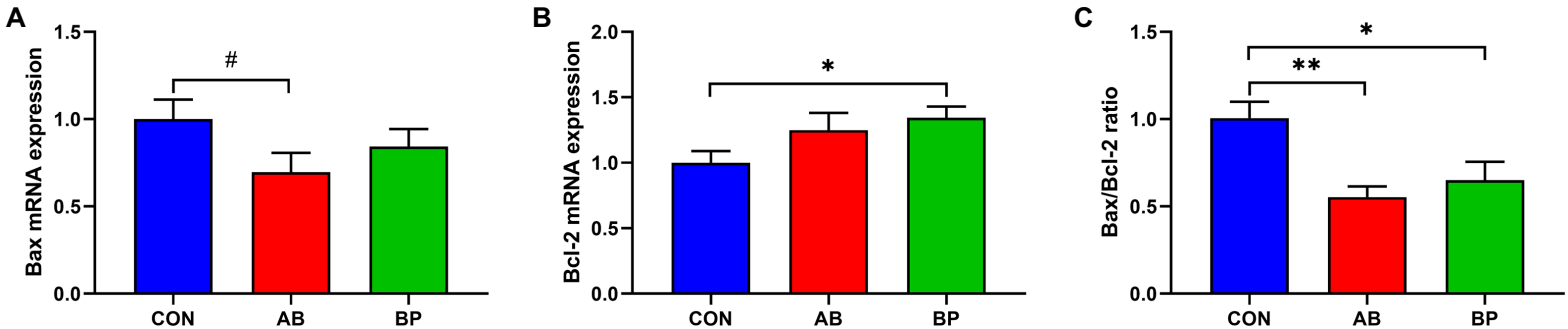
Effects of dietary supplemented with Bopu powder on expressions of apoptosis genes in intestine of broiler chickens. **(A)** Bcl-2 associated X (*Bax*); **(B)** B-cell lymphoma-2 (*Bcl-2*); **(C)** Bax/Bcl-2 ratio. CON group, broiler chickens fed a basal diet; AB group, broiler chickens fed a basal diet supplemented with 50 mg/kg aureomycin; BP group, broiler chickens fed a basal diet supplemented with 40 mg/kg Bopu powder containing protopine and allocryptopine. Values are mean ± standard error (*n* = 6). ^#^0.05 ≤ *p* < 0.10, *p < 0.05, **p < 0.01.

As shown in Figure 10, compared with CON group, BP group had significantly higher *Bcl-2* mRNA expression (*p* < 0.05), and BP group and AB group had significantly lower *Bax/Bcl-2* ratio (*p* < 0.05). Besides, BP group tended to had lower mRNA expression of *Bax* than CON group (*p* < 0.10).

### Effect of Bopu powder on intestinal microbial diversity of broiler chickens

**Figure 11:**
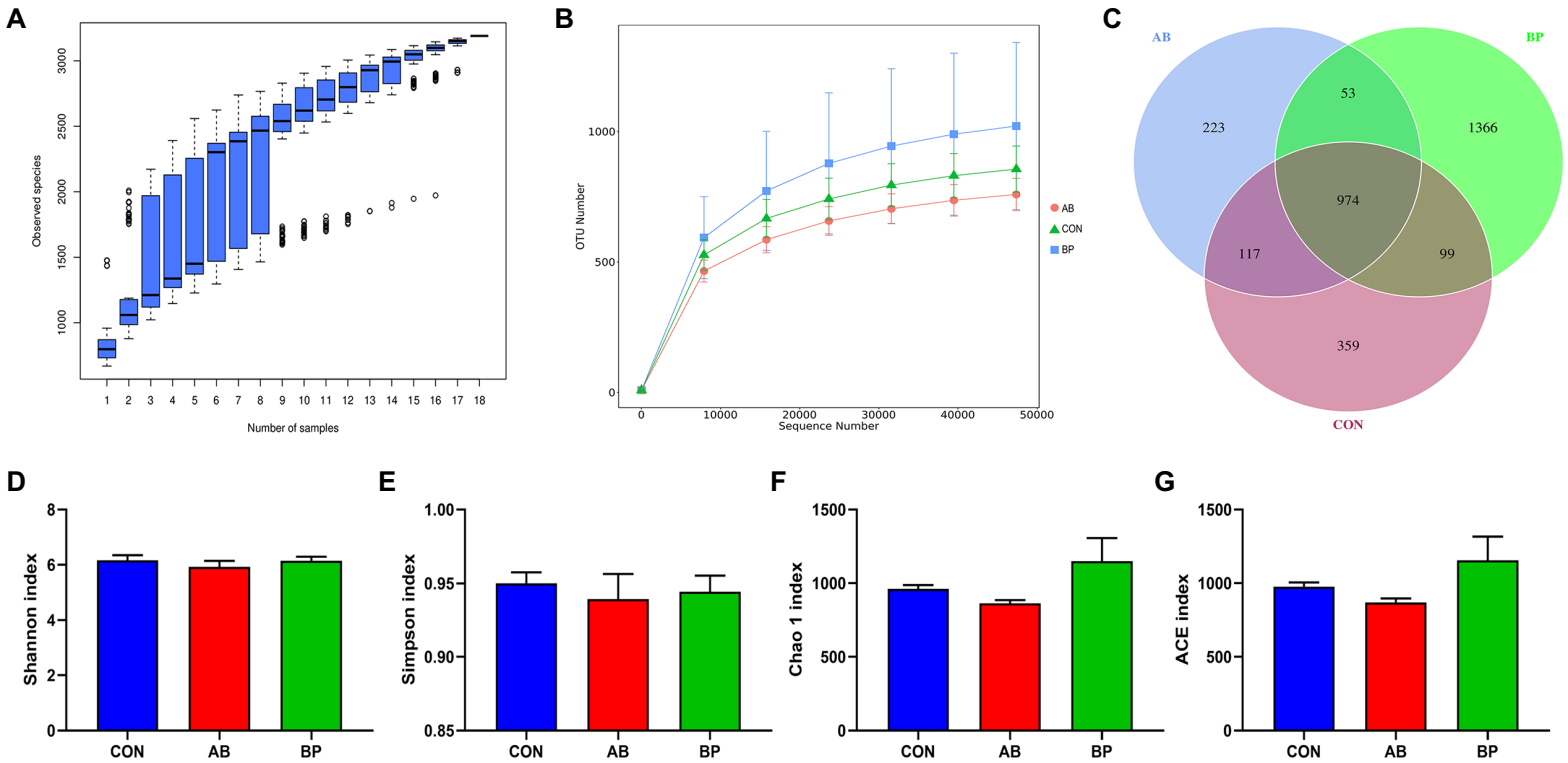
Differences in alpha diversity and richness among the three treatments. **(A)** The species accumulation curves; **(B)** The rarefaction curve of OTU; **(C)** Venn diagrams are displayed to describe the shared and unique sequences between processes; **(D)** Shannon index; **(E)** Simpson index; **(F)** Chao 1 index; and **(G)** ACE index. CON group, broiler chickens fed a basal diet; AB group, broiler chickens fed a basal diet supplemented with 50 mg/kg aureomycin; BP group, broiler chickens fed a basal diet supplemented with 40 mg/kg Bopu powder containing protopine and allocryptopine. *n* = 6. Differences between groups were considered significant at *p* < 0.05.

Sequence analysis based on the hypervariable region V4 of the 16S rDNA genes, the three groups obtained 1,097,479 total tags, including 1,024,789 taxon tags, 20 unclassified tags, and 72,670 unique tags. As shown in Figure 11A, the species accumulation curves tended to flatten as the number of analyzed sequences increased up to 18, indicating that our samples were sufficient for OTU testing and could predict the species richness of samples. Based on the OTU of 97% similarity, the rarefaction curve constructed (Figure 11B) tended to approach the asymptote, indicating that the sequence depth was also sufficient to represent most species richness and bacterial community diversity. As shown in Venn diagram (Figure 11C), the number of unique sequences was greatest (1,366) in the BP group and smallest (223) in AB group, for a total of 974 across the three treatment groups. However, there were no significant differences in Shannon (Figure 11D), Simpson (Figure 11E), Chao1 (Figure 11F), and ACE (Figure 11G) indexes among the three treatments (*p* > 0.05).

**Figure 12:**
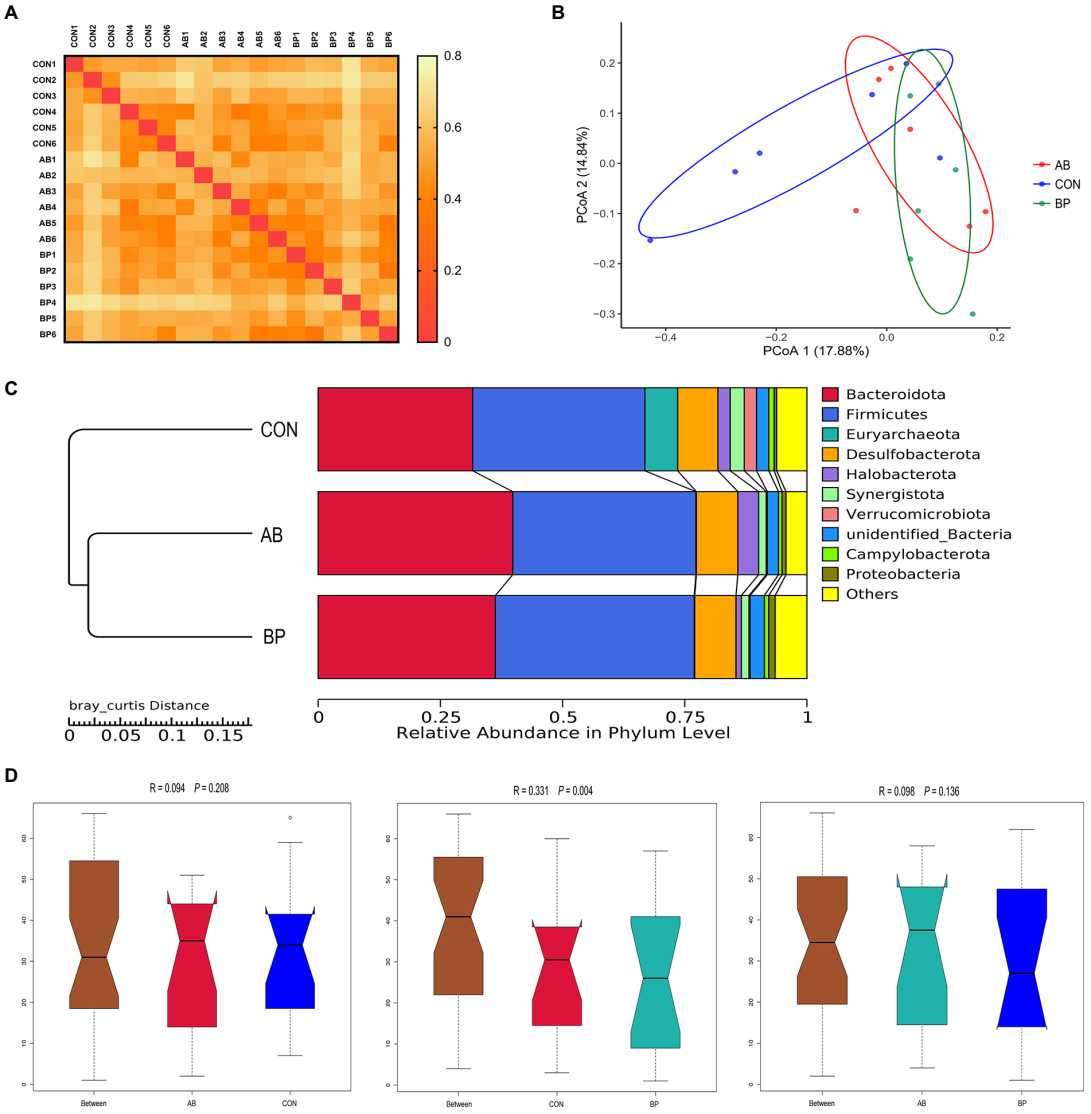
Beta diversity analysis of cecal community. **(A)** The results of the heat map drawn using bray_curtis distance matrix; **(B)** The principal coordinate analysis (PCoA); **(C)** Unweighted pair-group method with arithmetic mean (UPGMA) phylogenetic tree; and **(D)** The analysis of ANOSIM. CON group, broiler chickens fed a basal diet; AB group, broiler chickens fed a basal diet supplemented with 50 mg/kg aureomycin; BP group, broiler chickens fed a basal diet supplemented with 40 mg/kg Bopu powder containing protopine and allocryptopine. *n* = 6. Differences between groups were considered significant at *p* < 0.05.

In addition, the heat-map based on the bray_curtis distance matrix (Figure 12A) showed that the AB and BP group pairs had the minimum value, while the CON and the BP group pairs had the maximum value. The PCoA analysis (Figure 12B) revealed the BP samples dispersed far apart with the CON samples, suggesting a clear separation between the BP group and the CON group. Moreover, the UPGMA phylogenetic tree (Figure 12C) also showed that the AB and BP groups were close together and clustered in one group that exhibited the highest similarity, while the CON group was distributed in a separate branch, indicating that the CON group had a clearly different distribution from the other groups. Consistently, the ANOSIM (Figure 12D) showed that there was no significant difference (*p* > 0.05) in the microbial community structure between group CON and AB and group AB and BP pairs, while significant difference was observed in the bacterial community structure between the BP group and CON group (*p* < 0.05).

### Effect of Bopu powder on microbial relative abundance in cecum of broiler chickens

The relative abundances at the phyla level in cecal microbiota (top 10) are shown in Figure 12C. The most abundant phyla were found to be Firmicutes and Bacteroidetes, which accounted for 40.68 and 39.80%, respectively. There were no significant differences observed in relative abundances of the other phyla among the groups (*p* > 0.05, Supplementary Table S3).

Changes in relative abundance at the genus level (top 30) in broiler cecal microbiota are shown in Supplementary Table S4. The relative abundance of Firmicutes was contributed by *Phascolarctobacterium*, *Megamonas*, *Romboutsia*, *Limosilactobacillus*, *Ligilactobacillus*, *Erysipelatoclostridium*, *Butyricicoccus*, *Colidextribacter*, *NK4A214_group*, *UCG-005*, *CHKGl001*, and *Faecalibacterium*; Bacteroidota mainly distributed with *Alistipes*, *Prevotellaceae_UCG-001*, *Barnesiella*, *Bacteroides*, and *Parabacteroides*. Of the top 30 genera, compared with CON group, broiler chickens in the BP group had significantly higher (*p* < 0.05) relative abundances of *Faecalibacterium* and *Colidextribacter*.

### Correlation analysis between differential bacterial abundances and parameters in serum and intestine

**Figure 13:**
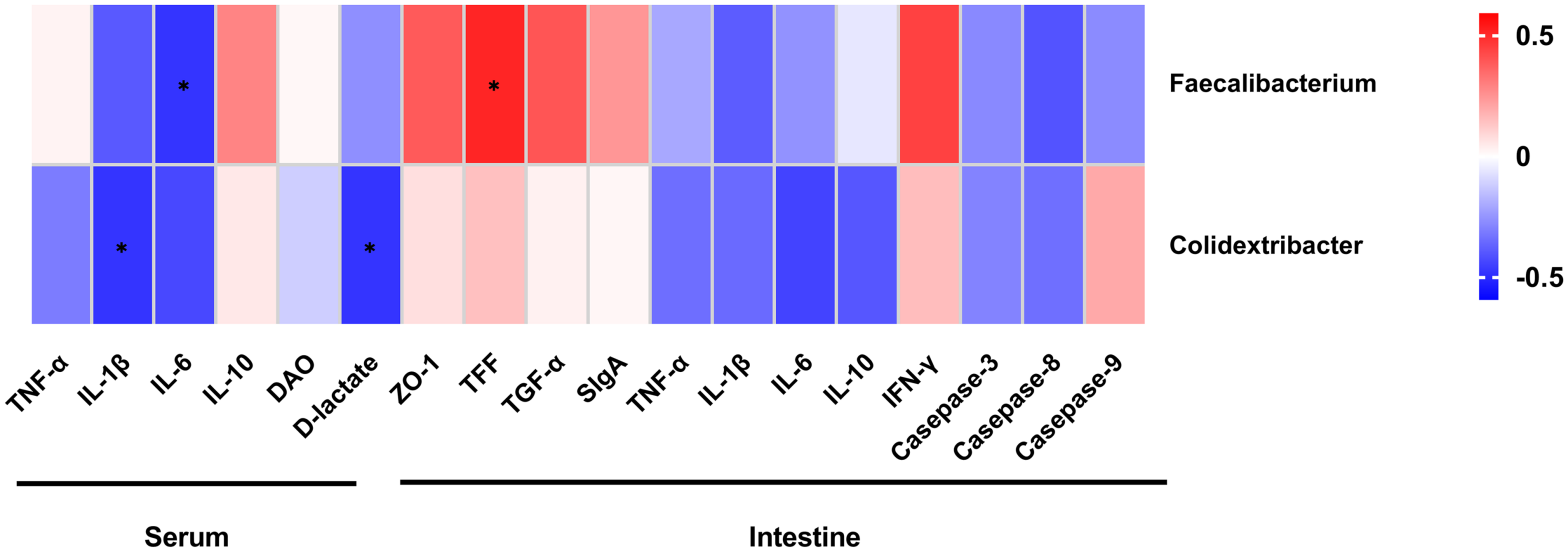
Spearman’s correlation analysis between differential bacterial abundances and concentrations of metabolic parameters and immunological markers in serum and intestine. Tumor necrosis factor-α (TNF-α); Interleukin-1β (IL-1β); Interleukin-6 (IL-6); Interleukin-10 (IL-10); zonula occludens-1 (ZO-1); Trefoil peptides (TFF); Transforming growth factor alpha (TGF-α); Secretory immunoglobulin A (SIgA); and Interferon-γ (IFN-γ). *The correlation is significant at a level of 0.05.

As shown in Figure 13, *Faecalibacterium* abundance was significantly positively correlated with intestinal TFF concentration (*r* = 0.511, *p* < 0.05), and negatively correlated with serum IL-6 concentration (*r* = −0.478, *p* < 0.05). *Colidextribacter* abundance had significant negative correlation with serum concentrations of IL-1β (*r* = −0.478, *p* < 0.05) and D-lactate (*r* = −0.478, *p* < 0.05).

## Discussion

Our current results showed that addition of Bopu powder to the diet increased villus height and VH to CD ratio. Larger villus height and higher VH to CD ratio indicate higher intestinal absorption efficiency of nutrients, which helps to improve growth performance of broilers (Chen et al., 2021a). In weaned piglets, dietary supplementation with 50 mg/kg MCE containing quaternary-benzo(c)phenanthridine alkaloids and protopine alkaloids increases VH and the VH to CD ratio (Chen et al., 2019). In the current study, addition of Bopu powder or aureomycin to the diet reduced serum DAO activity. Aureomycin treatment in rats also reduces intestinal DAO activity (Sato et al., 2016). Serum DAO activity is considered a marker of intestinal permeability which increases upon damage of the intestinal barrier (Luk et al., 1983; Wu et al., 2013). Moreover, we found that Bopu powder supplementation increased mucosal ZO-1, TFF, and TGF-α levels as well as *ZO-1* and *OCLN* mRNA levels in the small intestine of broiler chickens. Trefoil peptides (TFFs) are bioactive peptides, playing an important role in protecting against gastrointestinal injury and strengthening the intestinal barrier (Buda et al., 2012). Transforming growth factor-α has trophic effects on the intestinal epithelium and is helpful to maintain the integrity of intestinal epithelium cells (Schumacher et al., 2018). Peripheral membrane protein ZO-1 and transmembrane protein OCLN play important roles in maintaining intestinal tight junction integrity and mucosal barrier function (Zhang et al., 2017; Goo et al., 2019). In pigs, supplementation of the diet with isoquinoline alkaloids increases the expression of ZO-1 and enhances intestinal integrity (Ni et al., 2016). Our results indicated that dietary Bopu powder supplementation increased *GLUT2* expression in the small intestinal mucosa of broiler chickens. Elevated *GLUT2* mRNA levels typically contribute to intestinal digestive absorption (Yin et al., 2019).

Dietary Bopu powder supplementation decreased caspase-3, caspase-8, and caspase-9 activities in the small intestine of broiler chickens, and addition of aureomycin to the diet reduced intestinal caspase-3 and caspase-8 activities. Caspases are crucial in inflammation responses and cell death, and caspase-8 and caspase-9 can initiate the apoptotic process by activating the caspase-3, which initiates the process of apoptosis and induces cell death (Thornberry, 1997; Brentnall et al., 2013; D’Arcy, 2019). Further, Bopu powder supplementation increased *Bcl-2* mRNA and decreased the *Bax*/*Bcl-2* ratio in broiler chickens. Anti-apoptotic Bcl-2 and pro-apoptotic Bax play important roles in cell death, and high Bax/Bcl-2 ratio can also lead to the activation of caspase-3 (Reed et al., 1996; Korsmeyer, 1999). Taken together, our results suggest that Bopu powder supplementation can decrease apoptosis through inactivating caspase-3 through suppressing intrinsic or extrinsic pathways, which may in part explain the observed improvement of small intestinal development and mucosal barrier integrity.

Inflammation response is a major factor causing mucosal barrier damage and apoptosis (Sanchez de Medina et al., 2014). In the present study, Bopu powder addition reduced TNF-α, IL-1β, and IL-6 concentrations in the serum and small intestine of broiler chickens. These compounds are established markers of inflammatory responses (Akdis et al., 2016; Ducatelle et al., 2018), and our previous study showed that dietary Bopu powder reduced IL-1β and IL-6 levels in the liver of broilers (Liu et al., 2022a). In the current study, supplementation with aureomycin reduced serum TNF-α, IL-1β, and IL-6 concentrations and intestinal TNF-α and IL-6 concentrations, which is in line with the results of previous study (Song et al., 2022). Furthermore, dietary Bopu powder supplementation increased IL-10 concentrations in the serum and IFN-γ and SIgA concentrations in the intestinal mucosa of broiler chickens in the present study. IL-10 exerts anti-inflammatory and immunomodulatory effects (Kanai et al., 2015). Supplementation with 50 mg/kg MCE increases serum IL-10 concentrations in weaned piglets (Wang et al., 2021). The pleiotropic cytokine IFN-γ exerts antiviral, antitumor, and immunomodulatory functions (Jorgovanovic et al., 2020). Secretory immunoglobulin A is produced by plasma cells of the lamina propria on the surface of the mucosa, and increased mucosal SIgA is typically associated with increased defense against pathogenic bacteria (Goonatilleke et al., 2019). These results suggest that the protective effect of Bopu powder may be due to reduced pro-inflammatory stimulation and increased anti-inflammatory responses. Interestingly, dietary supplementation with Bopu powder or aureomycin reduced IL-10 concentrations in the small intestine of broiler chickens, which may be related to decreased levels of anti-inflammatory cytokines (Alfen et al., 2018). The TLR4/MyD88/NF-κB signaling pathway was evaluated with regard to inflammatory responses, and dietary supplementation with Bopu powder decreased *MyD88* and *NF-κB* mRNA levels in the intestines of broiler chickens, whereas aureomycin supplementation significantly decreased intestinal *NF-κB* mRNA levels. Previous studies illustrated that TLR4 can induce inflammatory responses through activating the NF-κB signaling pathway *via* the MyD88 protein (Hu et al., 2020). Inhibiting the TLR4/MyD88/NF-κB signaling pathway may be conductive to intestinal protection and inhibition of intestinal inflammation (Li et al., 2020; Yang et al., 2021).

Inflammatory responses are frequently associated with oxidative stress. In the present study, dietary Bopu powder supplementation increased *Sirt1*, *Nrf2*, *HO-1*, and *SOD1* mRNA levels in the small intestine of broiler chickens. Activation of Sirt1 can protect intestinal epithelium cells from oxidative injury *via* regulating Nrf2-related pathways (Chong et al., 2012). Nrf2 is a key transcription factor in antioxidant defense and can enhance the activity of HO-1, an important antioxidative enzyme regulating the levels of cellular ROS (Loboda et al., 2016; Zhuang et al., 2019). Superoxide dismutase is an important antioxidant enzyme removing free radicals (Chen et al., 2013), and its expression is upregulated through Nrf2 activation (Meng et al., 2018). Therefore, our current results suggest that Bopu powder supplementation can enhance intestinal antioxidant capacity in broiler chickens by activating the Sirt1/Nrf2 signaling pathway.

We observed significant differences in microbial community composition between BP and CON, and addition of Bopu powder in the diet increased cecal abundances of *Faecalibacterium* and *Colidextribacter*. *Faecalibacterium* is one of the major producers of butyrate in the intestine and exerts anti-inflammation functions through maintaining bacterial enzyme activity and deterring pathogens invasion (Miquel et al., 2013). *Faecalibacterium* abundance was positively correlated with intestinal TFF concentrations and negatively correlated with serum IL-6 concentrations in the present study. *Colidextribacter* can promote the production of inosine which helps reduce the secretion of inflammatory factors (Wang et al., 2019; Mager et al., 2020; Guo et al., 2021). In current study, *Colidextribacter* abundance was negatively correlated with serum IL-1β and D-lactate concentrations, suggesting that *Colidextribacter* abundance may help regulate systemic inflammatory responses and maintain integrity of the intestinal mucosa. Bopu powder supplementation thus affected intestinal bacterial communities and increased the abundance of beneficial bacteria, which may also explain the observed improved intestinal development.

## Conclusion

In conclusion, dietary supplementation with 40 mg/kg Bopu powder promoted intestinal development and function in broiler chickens through decreasing inflammatory response, enhancing antioxidant capacity, and increasing beneficial bacteria abundances. Besides, Bopu powder supplementation suppressed intestinal inflammatory response possibly through inhibiting TLR4/MyD88/NF-κB signal pathway and enhanced antioxidant capacity possibly by activating Sirt1/Nrf2 signal pathway in broiler chickens. Our study provides robust support for the application of Bopu powder as a potential substitute to AGP in improving intestinal health in poultry production.

## Data availability statement

The datasets presented in this study can be found in online repositories. The names of the repository/repositories and accession number(s) can be found at: https://www.ncbi.nlm.nih.gov/, PRJNA848931.

## Ethics statement

The animal study was reviewed and approved by the protocol for this experiment involving animals was in accordance with the guidelines of the Care and Use committee of Shandong Agricultural University (Ethics Approval code: SDAUA-2021-019).

## Author contributions

HL, NJ, WY, and YLi: conceptualization. YLiu, QW, and JN: data curation. YLiu, QW, and HL: formal analysis. WY and YLi: funding acquisition. QW, NJ, QG, and WY: investigation. YLiu, JN, NJ, LH, SJ, and YLi: methodology. YLiu, QW, and JN: project administration. JN, HL, and LH: resources. YLiu and QW: software. WY and YLi: supervision and writing—review and editing. SJ and YLi: validation. QW and WY: visualization. YLiu: writing—original draft. All authors contributed to the article and approved the submitted version.

## Funding

This research was funded by the Shandong Province Pig Industry Technology System (SDAIT-08-04 and SDAIT-08-05) and Postdoctoral Science Foundation of Shandong Agricultural University, grant number 040/76598.

## Conflict of interest

QW is employed by Shandong Wonong Agro-tech Group Co., Ltd. QG is employed by Shandong Landoff Biotechnology Ltd.

The remaining authors declare that the research was conducted in the absence of any commercial or financial relationships that could be construed as a potential conflict of interest.

## Publisher’s note

All claims expressed in this article are solely those of the authors and do not necessarily represent those of their affiliated organizations, or those of the publisher, the editors and the reviewers. Any product that may be evaluated in this article, or claim that may be made by its manufacturer, is not guaranteed or endorsed by the publisher.
