# Supplementary material for: Effects of dietary Bopu powder supplementation on intestinal development and microbiota in broiler chickens
Source: Front Microbiol. 2022 Oct 13;13:1019130. doi: 10.3389/fmicb.2022.1019130 (PMC9612830; doi:10.3389/fmicb.2022.1019130)
Supplement: Supplementary file 1 [file Data_Sheet_1.pdf]

Table S1. Ingredients composition and nutrient levels of basal diets (as-fed basis).

Items	Phases	
	**0-21 d**	**21-42 d**
Ingredients, %		
Corn	55.91	55.91
Soybean meal, 44% CP	13.78	10.18
Wheat bran	11.98	12.98
Corn starch residue	7.99	9.98
Corn gluten meal	3.99	3.99
Extruded soybean	1.50	2.10
Limestone	1.70	1.70
Calcium monophosphate	1.10	1.10
L-Lysine HCl, 76.8%	1.00	1.00
DL-Methionine, 98%	0.20	0.20
L-Threonine, 98%	0.10	0.10
Sodium chloride	0.40	0.40
Choline	0.10	0.10
Phytase	0.10	0.10
Complex enzyme	0.02	0.02
Trace mineral premix [1]	0.10	0.10
Vitamin premix [2]	0.02	0.02
Antioxidant	0.02	0.02

Total	100	100
Calculated analysis， %		
Metabolizable energy，MJ/kg	12.33	12.50
Crude protein	19.47	17.93
Crude fat	3.45	3.74
Calcium	0.94	0.87
Available phosphorus	0.35	0.33
Lysine	1.15	1.00
Methionine	0.50	0.40

[1] Provided per kilogram of complete basal diet: 10 mg of Cu as $CuSO_4$, 100 mg of Fe as $FeSO_4$, 1.1 mg of I as $Ca(IO_3)_2$, 65 mg of Zn as $ZnSO_4$, 100 mg of Mn as $MnSO_4$ and 0.3 mg of Se as Na_2SeO_3.

[2] Provided per kilogram of complete basal diet: vitamin A 10,000 IU, vitamin D_3 3,000 IU, vitamin E 30 IU, menadione 1.3 mg, thiamine 2.2 mg, riboflavin 8 mg, pyridoxine 4 mg, vitamin B_{12} 0.025 mg, D-biotin 0.2 mg, niacin 40 mg, folic acid 1 mg and D-calcium pantothenate 10 mg.

Table S2. Primer sequences used for quantitative real-time PCR.

Genes[b]	GenBank	Primer sequences, 5′-3′ [a]	Size, bp
β-actin	NM_205518.1	F:ATTGTCCACCGCAAATGCTTC<br>R:AAATAAAGCCATGCCAATCTCGTC	113
ZO-1	XM_015278981.2	F:CCACCTCAGAATAAGCCAGCAAT<br>R:CGGTTGTAAGAAGGAGTGACTGTT	146
OCLN	NM_205128.1	F:ATCAACGACCGCCTCAATCAG<br>R:TCCTCTGCCACATCCTGGTATT	83
CLDN2	NM_001277622.1	F:ACATTGGTTCAAGCATCGTGAC<br>R:GCTGTAGATGTCGCACTGAGT	101
CLDN3	NM_204202.1	F:GCCAAGATCACCATCGTCTCC<br>R:ATCACCAGCGGGTTGTAGAAAT	116
GLUT2	NM_207178.2	F:TTTCGAGAGAGCCGGTGTTG<br>R:GCCTTCTCCACCAGGAAGAC	102
SGLT1	NM_001293240.1	F:CATCGTTATCCTGGCAGTCTCCTT<br>R:TCATCGGGTTTCTCCTCCTCATCA	138
y+LAT1	XM_418326.5	F:CTCTCTCTCATCATCTGGGC<br>R: TCATTCCTGGGTCTGTTGCT	472
CAT1	NM_001145490	F:CTCTGGCTTGGTGGTGAACATCT<br>R:CGTGCTTGGCTTGAGGGTAGT	88
FABP1	NM_204192.4	F:ACTGGCTCCAAAGTAATGACCAATG	132

		R:TGTCTCCGTTGAGTTCGGTCAC	
TLR4	NM_001030693.2	F:CATCTCTGGAGTTCCTGCTGAA<br>R:TGTATGGATGTGGCACCTTGA	145
MyD88	NM_001030962.5	F:CGGAGGATGGTGGTCGTCATT<br>R:TCGTTCTTCATGGTCTTGCACTTG	140
NF-κB	NM_001396038.1	F:CAGCCCATCTATGACAACCG<br>R:TCAGCCCAGAAACGAACCTC	152
Sirt1	NM_001004767.1	F:CACGCCTTGCTGTAGACTTCC<br>R:ATGAACTTGTGGCAGAGAGATGG	148
Nrf2	MN416129.1	F:CACGCCTTGCTGTAGACTTCC<br>R:ATGAACTTGTGGCAGAGAGATGG	109
HO-1	NM_205344.2	F:GTCCCGAATGAATGCCCTTGA<br>R:ATGACCGTTCTCCTGGCTCTT	139
CAT	NM_001031215.2	F: GGAGGTAGAACAGATGGCGTATG<br>R: CGATGTCTATGCGTGTCAGGAT	114
SOD1	NM_205064.2	F:CGCAGGTGCTCACTTCAATCC<br>R:CAGTCACATTGCCGAGGTCAC	89
SOD2	NM_204211.2	F:GCTGTATCAGTTGGTGTTCAAGGA<br>R:GCAATGGAATGAGACCTGTTGTTC	130
GPX1	NM_001277853.3	F:CGGCTTCAAACCCAACTTCAC<br>R:CTCTCTCAGGAAGGCGAACAG	85
NQO1	NM_001277619.2	F:GAGTGCTTTGTCTACGAGATGGA	104

		R:ATCAGGTCAGCCGCTTCAATC	
Bax	XM_422067	F:TGAGCATGTAGCAACGGAAG<br>R:AGCAAGCTGATTGACGGTCT	295
Bcl-2	NM_205339.3	F:AGGACAACGGAGGATGGGATG<br>R:CACCAGAACCAGGCTCAGGAT	110

[a] F: forward primer; R: reverse primer

[b] ZO-1, zonula occludens-1; OCLN, occludin; CLDN2, claudin-2; CLDN3, claudin-3; GLUT2, glucose transporter type 2; SGLT1 , sodium-glucose transporter 1; y+LAT1, y+L amino acid transporter-1; CAT1, cationic amino acid transporter-1 ; FABP1, fatty acid binding protein-1; TLR4, toll-like receptor 4; MyD88, myeloid differentiation primary response 88; NF-κB, nuclear factor-kappa B; Sirt1, sirtuin1; Nrf2, nuclear factor erythroid 2-related factor 2; HO-1, heme-oxygenase 1; SOD1, superoxide dismutase 1; SOD2, superoxide dismutase 2; CAT, catalase; GPX1, glutathione peroxidase-1; NQO1, NAD(P)H quinone oxidoreductase 1; Bax, bcl-2 associated X; Bcl-2, b-cell lymphoma-2.

Table S3. Effects of Bopu powder extract supplementation in the diet on microbial relative abundance at phylum level (top 10) in cecal digesta of broiler chickens.

Items, %	Treatment [1]			SEM	*P* values		
	CON	AB	BP		CON *vs.* AB	CON *vs.* BP	AB *vs.* BP
Bacteroidota	31.64	39.80	36.27	2.54	0.219	0.440	0.613
Firmicutes	35.22	37.50	40.68	1.98	0.635	0.338	0.517
Euryarchaeota	6.74	0.06	0.11	1.54	0.148	0.151	0.475
Desulfobacterota	9.56	8.51	8.43	1.07	0.914	0.616	0.981
Halobacterota	2.50	4.25	1.13	0.63	0.330	0.236	0.069
Synergistota	2.90	1.56	1.57	0.47	0.322	0.340	0.998
Verrucomicrobiota	2.50	0.20	0.19	0.52	0.170	0.170	0.990
unidentified_Bacteria	2.53	2.34	2.94	0.21	0.723	0.380	0.324
Campylobacterota	1.09	0.72	0.88	0.22	0.503	0.757	0.734
Proteobacteria	0.35	0.79	1.30	0.24	0.594	0.120	0.473

[1] CON, broilers fed with a basal diet; AB, broilers fed with a basal diet supplemented with 50 mg/kg aureomycin; BP, broilers fed with a basal diet supplemented with 0.6 mg/kg Bopu powder (BP) containing protopine and allocryptopine.

Values are presented as mean and standard error of mean ($n = 6$). Differences between treatments were considered significant at $P < 0.05$.

Table S4. Effects of Bopu powder supplementation in the diet on microbial relative abundance at genus level (top 30) in cecal digesta of broiler chickens.

Items, %	Treatment [1]			SEM	*P* values		
	CON	AB	MCE		CON *vs.* AB	CON *vs.* BP	AB *vs.* BP
Bacteroides	5.79	11.96	11.54	2.59	0.372	0.323	0.955
Alistipes	16.74	17.11	17.16	1.24	0.889	0.904	0.990
Methanobrevibacter	6.74	0.06	0.11	1.54	0.148	0.151	0.458
Megamonas	2.65	0.55	6.15	1.28	0.367	0.363	0.113
Desulfovibrio	7.45	8.05	8.05	1.05	0.832	0.802	0.999
Faecalibacterium	4.12	5.24	8.15	0.89	0.621	0.043	0.213
Prevotellaceae_UCG-001	2.14	1.36	0.20	0.66	0.711	0.359	0.145
CHKCI001	1.36	2.80	1.28	0.61	0.449	0.892	0.429
[Ruminococcus]_torques_group	2.28	4.29	2.90	0.48	0.161	0.379	0.307
Methanocorpusculum	2.50	4.25	1.13	0.63	0.330	0.238	0.069
Synergistes	2.90	1.57	1.57	0.47	0.322	0.340	0.998
Akkermansia	2.46	0.19	0.10	0.53	0.148	0.164	0.550
Barnesiella	2.60	3.94	3.53	0.45	0.304	0.477	0.624
Parabacteroides	1.30	2.34	1.75	0.36	0.257	0.549	0.588
Ligilactobacillus	0.52	1.08	0.28	0.22	0.410	0.215	0.260
Phascolarctobacterium	2.15	2.32	1.77	0.15	0.665	0.184	0.176
Helicobacter	1.08	0.72	0.88	0.22	0.518	0.762	0.746

UCG-005	0.19	0.18	0.65	0.13	0.930	0.234	0.235
Limosilactobacillus	0.43	0.07	0.22	0.13	0.388	0.615	0.351
Bilophila	0.59	0.39	0.31	0.11	0.554	0.421	0.390
Romboutsia	0.98	1.41	0.87	0.12	0.181	0.674	0.091
Erysipelatoclostridium	0.42	0.52	0.75	0.11	0.621	0.275	0.490
Pseudomonas	0.00	0.32	0.01	0.11	0.364	0.172	0.377
unidentified_Erysipelotrichaceae	0.10	0.37	0.49	0.12	0.257	0.205	0.745
Colidextribacter	0.51	0.81	0.83	0.09	0.051	0.049	0.954
NK4A214_group	0.87	0.50	0.55	0.10	0.210	0.272	0.769
Olsenella	0.42	0.04	0.05	0.09	0.211	0.216	0.879
Butyricicoccus	0.36	0.75	0.43	0.08	0.110	0.607	0.172
Fusobacterium	0.09	0.02	0.13	0.03	0.214	0.633	0.110
Cerasicoccus	0.03	0.00	0.00	0.01	0.361	0.350	0.363

[1] CON, broilers fed with a basal diet; AB, broilers fed with a basal diet supplemented with 50 mg/kg aureomycin; BP, broilers fed with a basal diet supplemented with 0.6 mg/kg Bopu Powder (BP) containing protopine and allocryptopine.

[2] Values are presented as mean and standard error of mean (n = 6). Differences between treatments were considered significant at $P < 0.05$.
